# On Traumatic Spasms

**Published:** 1853-01

**Authors:** W. Colles


From Dublin Quarterly Journal.

*On Traumatic Spasms, by* W. Colles, Esq.

Mr. Colles describes four kinds of spasms occurring after injuries:

1. Immediately after the receipt of any injury, before any adjustment is attempted, the muscles of the limb are thrown into spasmodic action by the least motion, or by the handling of the parts. The treatment is merely to place the limb so that the muscles may be relaxed, to reduce the fracture, and apply firm but not too tight dressings. A full opiate will be useful, and sometimes should be preceded by the removal of a few ounces of blood. This is never a formidable affection, and seldom lasts beyond the second or third day.

2. This is the most severe and rapidly fatal form. About the third or fourth day after the injury, the patient, whenever he is dozing to sleep, is suddenly awakened by a severe jerk in the limb, and a very acute but transient attack of pain. These spasms, at first at irregular and distant intervals, become more frequent and regular; then the disease attacks the muscles of that side of the body, and extends gradually to the other side, till at length every voluntary muscle is in violent action, during this momentary spasm. The pulse becomes considerably increased in quickness, but not in force; the temperature is not increased at first; as the disease advances, a perspiration breaks out, and becomes more profuse, cold, and clammy towards the end, when the patient's mind begins to wander. Death generally occurs, apparently from exhaustion, between the second and sixth day from the invasion of the disease. Early amputation is believed by Dr. Colles to be the only remedy which can be depended on. After death, it is generally found that a portion of nerve is impacted between the ends of the bone, so that it is pressed on and inflamed.

3. The third form is that denominated tetanus, and which comes on towards the cure of the injury. On this form amputation has no influence, and it was probably from success in some cases of the second form which induced Baron Larrey to recommend amputation in tetanus. The diagnosis of the second and third form is important, and their more essential differences are thus given:

| | |
|---|---|
| 1. The second form of spasm comes on in three or four days after the accident. | Tetanus seldom appears before the second or third week. |

| | |
|---|---|
| 2. It commences by spasm in the limb injured. | Tetanus commences by stiffness of the (jaws and) throat. |
| 3. In the intervals between the spasms the muscles are much relaxed, and the patient can swallow and move with comparative ease. | In tetanus there is constant rigidity, almost preventing swallowing, or moving of any description, and giving a peculiar expression of countenance. |
| 4. The pain is chiefly in the wound, and is most excruciating during spasm. | In tetanus there is no pain in the wound, but a pain at the scrobiculus cordis. |
| 5. The disease runs its course in three or four days. | Tetanus may continue for as many weeks. |
| 6. Amputation holds out the only prospect of relief. | In tetanus, amputation is useless, if not injurious. |
| 7. The disease seems to have a local origin. | Tetanus is more of a constitutional affection. |

4. Mr. Colles next makes a few remarks on the affection, which results from implication of a nerve in a tight cicatrix. Division of the nerve, or amputation, can alone effect the complete removal of the disease.

